# Healthcare Utilization and All-Cause Premature Mortality in Hungarian Segregated Roma Settlements: Evaluation of Specific Indicators in a Cross-Sectional Study

**DOI:** 10.3390/ijerph15091835

**Published:** 2018-08-24

**Authors:** János Sándor, Anita Pálinkás, Ferenc Vincze, Nóra Kovács, Valéria Sipos, László Kőrösi, Zsófia Falusi, László Pál, Gergely Fürjes, Magor Papp, Róza Ádány

**Affiliations:** 1 Department of Preventive Medicine, Faculty of Public Health, University of Debrecen, Kassai St 26/B, H-4028 Debrecen, Hungary; palinkas.anita@sph.unideb.hu (A.P.); vincze.ferenc@sph.unideb.hu (F.V.); kovacs.nora@sph.unideb.hu (N.K.); sipos.valeria@sph.unideb.hu (V.S.); adany.roza@sph.unideb.hu (R.Á.); 2 Department of Financing, National Health Insurance Fund, Váci Rd 73/A, H-1139 Budapest, Hungary; korosi.l@neak.gov.hu (L.K.); falusi.zs@neak.gov.hu (Z.F.); pal.l@neak.gov.hu (L.P.); 3 National Institute for Health Development, Diószegi St 64, Budapest H-1113, Hungary; furjes.gergely@sph.unideb.hu (G.F.); papp.magor@nefi.hu (M.P.); 4 MTA-DE-Public Health Research Group, Department of Preventive Medicine, Faculty of Public Health, University of Debrecen, Kassai St 26/B, H-4028 Debrecen, Hungary; 5 WHO Collaborating Centre on Vulnerability and Health, Department of Preventive Medicine, Faculty of Public Health, University of Debrecen, Kassai St 26/B, H-4028 Debrecen, Hungary

**Keywords:** Roma minority, legal constraints, healthcare utilization, health status, geographical inequality

## Abstract

Roma is the largest ethnic minority of Europe with deprived health status, which is poorly explored due to legal constrains of ethnicity assessment. We aimed to elaborate health indicators for adults living in segregated Roma settlements (SRS), representing the most vulnerable Roma subpopulation. SRSs were mapped in a study area populated by 54,682 adults. Records of all adults living in the study area were processed in the National Institute of Health Insurance Fund Management. Aggregated, age-sex standardized SRS-specific and non-SRS-specific indicators on healthcare utilization and all-cause premature death along with the ratio of them (RR) were computed with 95% confidence intervals. The rate of GP appointments was significantly higher among SRS inhabitants (RR = 1.152, 95% CI: 1.136–1.167). The proportion of subjects hospitalized (RR = 1.286, 95% CI: 1.177–1.405) and the reimbursement for inpatient care (RR = 1.060, 95% CI: 1.057–1.064) were elevated for SRS. All-cause premature mortality was significantly higher in SRSs (RR = 1.711, 1.085–2.696). Our study demonstrated that it is possible to compute the SRS-specific version of routine healthcare indicators without violating the protection of personal data by converting a sensitive ethical issue into a non-sensitive small-area geographical analysis; there is an SRS-specific healthcare utilization pattern, which is associated with elevated costs and increased risk of all-cause premature death.

## 1. Introduction

Partly because their health status is considered deprived, Roma people are among the most vulnerable groups of Europe [1]. The Roma vs. non-Roma gap in health is assumed to be attributable to their poor living condition, high exposure to health risks, and problems with access to high-quality healthcare [2].

Although the anecdotal conclusions from the common experience of healthcare and public health professionals are supported by an increasing amount of research results, the lack of knowledge in this field remains significant [2,3,4,5,6]. In these circumstances, the necessary changes can be initiated neither through the organization of health services nor by enhancing the ability of Roma to use the health services properly [7]. Moreover, this problem hindering the elaboration of targeted interventions is reflected in the restricted effectiveness of programs that aim at improvement of the health status of Roma people in European countries with significant Roma minorities. Since Roma is the largest ethnic minority of Europe [8], the poor health status of the Roma people is among the most important equity issues in Europe.

The most important limitation on Roma studies may be the lack of a robust methodology to identify Roma people in health and demographical statistics and in epidemiological investigations [9]. A methodological development is therefore important to improve the quality of health status assessment and monitoring changes induced by different interventions.

The National Institute of Health Insurance Fund Management (NIHIFM) in Hungary maintains a nationwide primary healthcare monitoring system. A set of performance indicators that focuses on critical processes of long-term care is produced every month for all Hungarian general medical practices (GMPs). The reported results influence the financing of GMPs. This is the only official quality evaluating system in Hungarian healthcare. Although, in addition to the long-term care indicators, the NIHIFM regularly evaluates pathway indicators in relation to the use of secondary care services (provided by outpatient, inpatient and imaging centers) and primary healthcare provisions by adults who belong to a GMP, as well as the all-cause mortality rates by GMPs, these indicators are not utilized in practice financing.

In the NIHIFM data handling system, it is technically feasible to create healthcare indicators for any group of subjects if the identification of persons of the target group can be carried out without violating the right to personal data protection, if the necessity of the indicators is established by epidemiological data based on health needs, and if the health policy formulation requires the information. The geographical inequalities and the institutional performance indicators’ variability is routinely investigated in this system since persons living in a geographical area or cared for by an institution can be identified. Within the internal and safe IT system of NIHIFM, the data processing of person-level health records can be carried out to produce aggregated indicators. Aggregated indicators from this approach have been routinely applied for decades in Hungary. Convincing experiences exist regarding the safety and usefulness of this approach [10,11,12,13,14]. The NIHIFM facilities can, therefore, be used to produce Roma-specific healthcare indicators for regular monitoring, with identification of Roma persons under legal circumstances.

In Hungary, approximately one-quarter of the Roma people live in segregated Roma settlements (SRSs), where most inhabitants have Roma identity [15]. Because the SRS is a geographical concept, aggregated statistics can be generated for the population living there. By this approach, the most vulnerable part of the Roma community can be described by routine health indicators [16]. The critical point of this approach is the linkage between a database that describes the settlements geographically and the database of persons having health insurance through the addresses of residential places. If this link can be carried out in a safe way, then this approach can transform the task of Roma-specific indicator production into geographical inequality analysis.

Since Roma-specific routine health statistics have not yet been elaborated in Hungary, we undertook the adoption of existing, general population-targeted routine health statistics of NIHIFM by restricting the target population to adults living in SRSs. This project aimed (1) to elaborate an epidemiological, IT, and legal system that can produce Roma-specific primary healthcare indicators on healthcare utilization and all-cause premature mortality, applicable in the regular health statistical system and (2) to evaluate by these indicators the healthcare utilization and all-cause premature mortality of Roma people living in segregated settlements in comparison to the health status of other people who are living nearby, in the same villages and cities, provided by the same GPs, and have the same health service availability as Roma people.

## 2. Materials and Methods

### 2.1. Setting

This study investigated adult persons who were at least 18 years old, living in the intervention area of the Public Health Focused Model Program for Primary Care Development (PH–PC) in Hungary. The PH-PC was to elaborate and test new structures of primary healthcare by which the preventive services can be delivered by GPs and their co-workers in a way effective both for the general population and among Roma people. Details on the program have been published elsewhere [17,18,19,20].

### 2.2. Design

Our presented cross-sectional investigation was a secondary data analysis without primary data collection. The intervention area of the PH–PC was populated by 54,682 adults (7298 of them were Roma according to the baseline survey based on self-declaration of ethnicity) provided by 25 GPs [20]. Because the only health insurance institution in Hungary is the NIHIFM, data for all adults living in the intervention area were available and used for analysis, without any selection. The study used the database of NIHIFM from 2013, and produced the baseline reference data for PH–PC evaluation.

### 2.3. Mapping Segregated Roma Settlements

The primary data collection for mapping SRSs in the intervention area was carried out by the PH–PC. All the SRSs have been mapped in the towns and villages of the study area using a method described in detail elsewhere [15] and applied in different Roma surveys [16,21,22]. Briefly, the SRS was defined as part of a settlement with at least four residential units having lower housing and environmental quality than other parts of the same settlement. According to previous studies, 94% of the population determined by this approach is Roma [15]. Finally, all the residential units (addresses by town/village, street and number) of the studied settlements were classified as SRS or non-SRS households.

### 2.4. Roma Settlement Specific Version of NIHIFM’ Routine Primary Adult Care Indicators

The data for indicator computations were derived from the NIHIFM. There are separate indicator sets for children (aged less than 18 years) and for adults in the NIHIFM. Our study was confined to the adult care. Pathway, performance and all-cause premature mortality indicators [23] were computed for GMPs (Table 1).

The SRS-specific version of these indicators was computed within the NIHIFM. First, the adults cared for by the GPs of the study area were selected based on their health insurance identifier and address. Using the addresses from the SRS mapping sub-project, the SRS and the non-SRS adults were separated from each other. The age- (applied age groups: 18–24, 25–29, 30–34, 35–39, 40–44, 45–49, 50–54, 55–59, 60–64, 65–69, 70–74, 75–79, 80–X) and sex-specific nominators and denominators of the above–listed indicators were computed for the SRS and the non-SRS subsamples. Next, for descriptive purpose, the crude indicators were calculated for SRS and for non-SRS subsamples.

### 2.5. Statistical Analysis

The indicators were standardized by age and sex. The national age- and sex-specific reference values were used in the indirect standardization. The ratio of SRS-specific and non-SRS-specific standardized indicators (relative service utilization and mortality rates) were computed. These relative risk measures (RR) with their corresponding 95% confidence intervals (95% CI) have been applied to describe the relative riskiness of residents in SRSs. The statistical nature of the difference between indirectly standardized SRS-specific and non-SRS-specific indicators was evaluated by 95% CIs.

**Figure 1:**
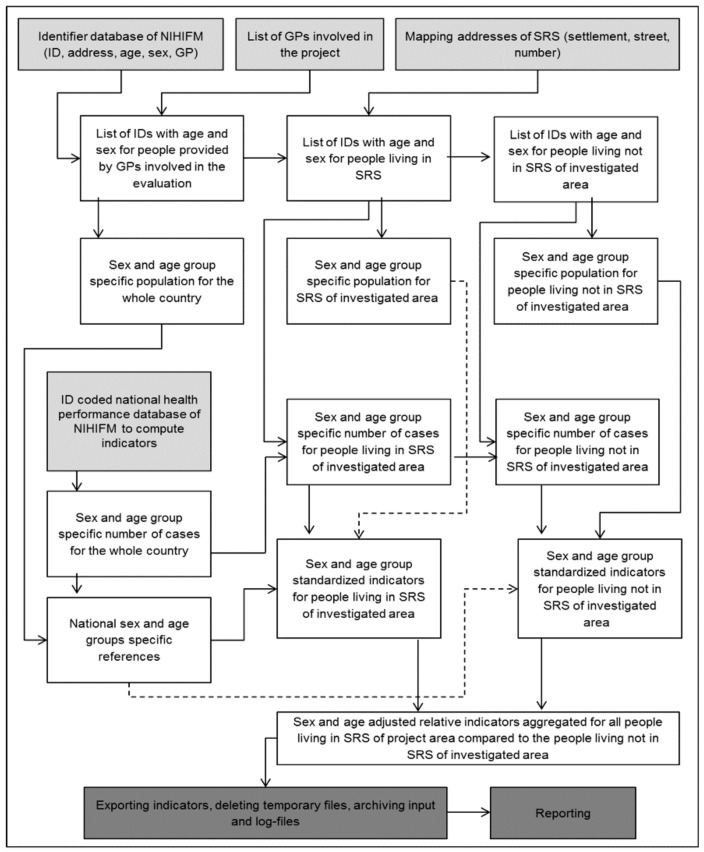
Data processing to produce routine Roma-specific primary care indicators for adults (by utilizing available administrative databases of the National Institute of Health Insurance Fund Management with avoiding person-level identification of Roma people; light gray-shaded input data; dark gray-shaded output data).

At the end of the data processing, the log files had been archived and the temporary files had been deleted, according to the internal rules of the NIHIFM. The standardized indicators aggregated for all studied SRSs and for all studied non-SRS areas and the relative risk measures were utilized for further analyses (Figure 1).

All statistical computations were performed using SPSS 18 (SPSS package for Windows, Release 18; SPSS, Chicago, IL, USA).

### 2.6. Ethics Approval and Consent to Participate

No person-level data collection was deployed in the study. Therefore, written informed consent was not required from members of the population investigated. The data collection to describe the geographical location of SRS was approved by the Research Ethics Committee of the Medical Research Council of Hungary (8907-0/2011-EKU, 285/PI/11 and 2213-5/2013/EKU, 233/2013). The protocol of the study was investigated and approved by the Office of the Commissioner for Fundamental Rights (AJB-3147/2013), the general director National Institute of Health Insurance Fund Management (E0101/215-3/2014), and the Hungarian National Authority for Data Protection and Freedom of Information (NAIH/2015/826/7N).

### 2.7. Availability of Data and Material

The database of aggregated statistics prepared for analysis is stored in secure, confidential, password protected storage in the server of the Debrecen University Faculty of Public Health. Completely de-identified records could be made available to interested persons/organizations on request to the corresponding author at sandor.janos@sph.unideb.hu.

## 3. Results

**Figure 2:**
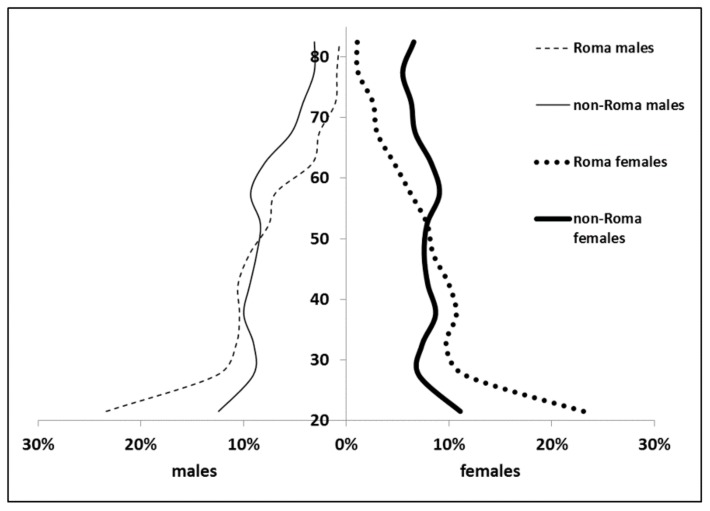
Distribution of age groups in the studied Roma and non-Roma populations.

The investigated area consisted of 16 villages and towns populated by 54,682 adults (26,117 males; 28,565 females). There were 16 identified SRSs, where 3022 adults (1519 males; 1503 females) resided. The demographic composition of SRS and complementary non-SRS areas deviated from each other profoundly (Figure 2). The mean age was much less in SRSs (total: 39.2 years, males: 38.6 years, females 39.8 years) than in non-SRSs (total: 48.4 years, males: 46.3 years, females: 50.2 years). Similarly, the old-age dependency ratio was considerably lower in SRS (total: 7.0%, males: 5.6%, females: 8.4%) than in non-SRS (total: 26.0% years, males: 18.75%, females: 33.5%).

### 3.1. Pathway Indicators

The crude pathway indicators for the study population are shown in Table 2.

The healthcare use pattern of SRS inhabitants differed significantly from that of non-SRS inhabitants according to the age- and sex-standardized relative indicators. The number of GP appointments per person per year was significantly higher among SRS inhabitants (RR = 1.152, 95% CI: 1.136–1.167). There was no observed difference between SRS and non-SRS with respect to the proportion of subjects receiving outpatient care per year. However, the number of interventions (RR = 0.893, 95% CI: 0.886–0.899) and the reimbursement for interventions (RR = 0.920, 95% CI: 0.914–0.927) in outpatient care per person per year was significantly reduced for SRS inhabitants. Although the use of imaging examinations was higher in SRSs (RR = 1.064, 95% CI: 1.011–1.120), the number of and reimbursement for imaging examinations were significantly reduced (RR = 0.895, 95% CI: 0.887–0.904, RR = 1.036, 95% CI: 1.025–1.047, respectively). The proportion of subjects hospitalized (RR = 1.286, 95% CI: 1.177–1.405), the duration of hospitalization (RR = 1.168, 95% CI: 1.138–1.200), and the reimbursement for inpatient care (RR = 1.060, 95% CI: 1.057–1.064) were elevated in SRSs.

### 3.2. Performance Indicators

The crude performance indicators for the study population are summarized in Table 3.

According to the age- and sex-standardized relative risk measures, most of the performance indicators show no difference between SRS and non-SRS areas. However, the Roma people above 65 years of age are less intensively vaccinated against influenza (RR = 0.675, 95% CI: 0.468–0.973), less frequently examined by ophthalmologists in case of diabetes (RR = 0.693, 95% CI: 0.493–0.975), less frequently treated with antibiotics (RR = 0.781, 95% CI: 0.718–0.850), and less frequently screened for breast cancer (RR = 0.381, 95% CI: 0.304–0.478).

### 3.3. All-Cause Premature Mortality

The crude all-cause premature mortality rate was 0.71% in SRS and 0.62% in non-SRS subsamples (number of registered death was 20 in SRS and 258 in non-SRS). The age- and sex-standardized all-cause premature mortality proved to be significantly higher in SRSs (RR = 1.711, 95% CI: 1.085–2.696).

## 4. Discussion

### 4.1. New Indicator Set for Segregated Roma Settlements

Our study demonstrates that it is possible to produce an SRS-specific version of the routine primary healthcare indicators of NIHIFM by utilizing the existing IT and solving legal problems by elaborating data processing technology, which ensures the complete protection of personal data throughout the whole process. This system utilizes healthcare use data from routine health statistics, which prevents the generation of significant expenses related to monitoring. The SRS geographical description was determined as a method that respected the right for personal data protection and was approved by a responsible ethics committee. The linkage between geographical data on SRS localization and the internal healthcare use databases of the NIHIFM was established according to the internal, safe and controlled protocols of the NIHIFM. The final products of the data processing, the aggregated indicators of SRS versus non-SRS healthcare use, cannot be linked to any person.

On the other hand, the observed indicators may be used to explore the specific problems of a vulnerable population with significant size and consequently with a significant impact on the health gap between Roma and non-Roma people. These indicators seem to have high reliability and high feasibility for application in routine monitoring that supports the elaboration and implementation of Roma-specific interventions. Using these indicators, improvement in the effectiveness of the Roma projects is expected. The necessity of this kind of monitoring technology has been emphasized by the EU, the WHO, and national authorities, as well [5,6,7,10,24].

### 4.2. Main Findings and Concordance with Others’ Observations

The quality of care for adults living in SRSs seems to not deviate from the reference of non-SRS areas in many respects. Cervical cancer screening, prevalence of diabetes and hypertension, serum creatinine, lipid and HbA1c laboratory investigations and the application of beta-blockers among patients with ischemic heart diseases are utilized with similar frequency in SRSs and non-SRSs. However, there are some services underutilized in SRSs, such as breast cancer screening, ophthalmologic check-up among diabetes patients, influenza vaccination, and use of antibiotics. There is no performance indicator that suggests that the utilization of services is better in SRS areas than in non-SRS areas.

Adults living in SRSs use primary healthcare more intensively. The same proportion of adults living in SRSs use the services of outpatient institutions as non-SRS adults, but the number of interventions and the amount of reimbursement is significantly less for them. Taking into consideration their worse health status and higher needs, services of outpatient institutions seems to be underutilized by SRS inhabitants.

A higher proportion of SRS adults use hospital care and imaging services. This may be a consequence of the worse health status and higher needs, but it may also be due to the underprovided secondary outpatient care, which omits the prevention of the underlying chronic disease progression. Per capita reimbursements are significantly higher for SRS inhabitants both in the respect of hospital and imaging care use.

Since most of the secondary care (out- and inpatient services, imaging) costs are generated by hospitals, which are used more intensively by SRS adults, and the primary healthcare is used more frequently by SRS inhabitants too, the resource needs of adults living in the SRS are higher than that of adults in the non-SRS.

The poor health status in SRSs is demonstrated by the profoundly higher all cause premature mortality in SRSs. This may reflect either the lower quality of healthcare or the worse health status caused by unhealthier life styles or disadvantageous socio-economic status [16,21]. The exploration of the association between poor quality of care and all-cause premature death needs further investigation. The value of the methodology used in this paper can be emphasized by the fact that due to the prohibition of registration of ethnicity in death certification, there are no Roma-specific mortality data in Hungary at all. Our observation is the first Roma-specific mortality figure in the country.

Our observations of Roma-specific healthcare utilization pattern are in concordance of published results of Roma studies from European countries, which were based on self-declared Roma ethnicity assessment [25,26,27,28,29,30]. The elevated risk of all-cause premature death among Roma adults living in SRS is also consistent with the Hungarian observations of the poor health status and poor self-rated health of adults living in SRS [16,21,22].

### 4.3. Strengths and Limitations

The approach presented in this paper may not have directly investigated the Roma health status. Instead, it evaluates the health status of adults living in SRSs in comparison to the non-SRS population. Consequently, this methodology is not a universal approach; it is not a final solution to monitor the Roma health status in general. However, this methodology can investigate the most disadvantageous stratum of the Roma population, which probably has the worst health status, thus requiring the most urgent interventions.

Since there are Roma adults not living in SRSs who have inferior health status, there is a misclassification introduced into the study that results in dilution of the relative risk measures. The observed risk elevations are underestimations of the real SRS effect. It is also probable that there were some outcomes that showed no significant association with the SRS in spite of the existence of the SRS influence, due to this bias that seemed to counterbalance the relatively small SRS impact. The higher the proportion of Roma individuals living in non-SRSs is, the more important are the above-mentioned effects (and the more underestimated are the problems among Roma that could be identified).

Because the adulthood is usually started earlier in Roma communities than in general Hungarian population, the health problems related to or originated from the early phase of adulthood (e.g., early motherhood) cannot be evaluated by our present approach.

Because not all but 94% of inhabitants of SRS belong to the Roma ethnic minority [15], the presented observations are approximations of Roma healthcare utilization and all-cause premature mortality. However, it is highly probable that the lifestyle and health behavior of non-Roma inhabitants of SRS are very similar to those of Roma living in SRS. Consequently, the presented indicators are good approximations of Roma health status.

### 4.4. Implications

The importance of SRS-focused interventions is accepted by Hungarian governmental institutions. The National Social Inclusion Strategy (national strategy for supporting socially vulnerable groups) as a governmental decree [24] defines the segregated settlements and makes it possible to map the settlements as a part of the routine social statistical services of the Hungarian Central Statistical Office. The SRS mapping carried out in our investigation as a part of PH-PC can be replaced by regular governmental activity. It establishes the possibility that the SRS-specific primary healthcare indicators demonstrated by this paper could be used in routine government-operated health monitoring. The proposed method is able to produce a monitoring tool similar to the self-reported ethnicity-based health statistical systems, which are far from perfect but very useful in the exploration and management of ethnicity-related problems, according to previous studies [31,32,33,34,35,36].

This proposed monitoring cannot evaluate the interrelationship between pathway, performance and all-cause premature mortality indicators, and the determinants of these indicators. Therefore, analytic investigations on determinants of healthcare utilization (applying the operationalized concept of utilization as realized access to healthcare [37]) among adults from SRS can be complementing in addition to the regular application of standardized SRS-specific indicators. Co-ordinated application of the SRS-specific indicators and the analytic investigations can support both the health policy agenda setting (identification of problems, prioritization) and the policy evaluation.

## 5. Conclusions

Our investigation demonstrates that it is possible to produce specific primary healthcare indicators for Roma adults living in SRSs in a way that fits the routine health statistical processes without violating the personal data protection laws and without generating significant expenses by converting a sensitive ethical issue to a non-sensitive small area geographical inequality analysis. Consequently, this indicator set can be applied in routine monitoring in Eastern and Central European countries where the legal system is similar to that of Hungary and where a significant proportion of Roma people are living in segregated settlements. According to results from our indicator development project, although there are some primary care services provided for the Roma as is done for non-Roma adults, many services are underutilized and some services are over-utilized by SRS inhabitant adults. Overall, there is a specific health service utilization pattern of adults living in SRS—although the cost is relatively more than for non-Roma people, which can contribute to the very high all-cause premature mortality of adults living in SRSs.

## Figures and Tables

**Table 1:** Pathway, performance and all-cause premature mortality indicators for adults living in segregated Roma settlements (SRSs) and living in the same municipalities’ complementary areas (non-SRSs) with the age- and sex-standardized relative rates (RRs) in care for inhabitants of SRSs.

Indicator Type	Indicator Name
pathway indicators	number of GP appointments per person per year
	proportion of subjects receiving outpatient care per year
	number of interventions in outpatient care per person per year
	reimbursement for interventions in outpatient care per person per year
	proportion of subjects hospitalized per year
	duration of hospitalization in inpatient care per person per year
	reimbursement for inpatient care per person per year
	proportion of subjects having an imaging examination per year
	total number of imaging examinations per person per year
	reimbursement for imaging examination in outpatient care per person per year
performance indicators	proportion of patients above 65 years of age vaccinated against influenza within the last 12 months
	proportion of patients aged 40–54 years with treated hypertension (taking antihypertensive medication at least four times within 12 months)
	proportion of patients aged 55–69 years with treated hypertension (taking antihypertensive medication at least four times within 12 months)
	proportion of patients having a serum creatinine test within the last 12 months among treated hypertensive patients (taking antihypertensive drugs at least four times within the last 12 months)
	proportion of patients having a lipid status assessment within the last 12 months among treated hypertensive and/or treated diabetes patients (taking antihypertensive medication at least four times within 12 months and/or taking ATC code A10 drugs at least four times within the last 12 months)
	proportion of patients taking beta-blocker medication at least four times within 12 months relative to the total number of acute myocardial infarction (MI) and/or coronary artery bypass surgery (CABG) and/or percutaneous coronary intervention (PTCA) patients
	proportion of patients among treated diabetes patients having a hemoglobin A1c test within the last 12 months (taking ATC code A10 drugs at least four times within the last 12 months)
	proportion of patients among treated diabetes patients examined by ophthalmologist within the last 12 months (taking ATC code A10 drugs at least four times within the last 12 months)
	proportion of patients aged 40–54 years with treated diabetes mellitus (taking ATC code A10 drugs at least four times within 12 months)
	proportion of patients aged 55–69 years with treated diabetes mellitus (taking ATC code A10 drugs at least four times within 12 months)
	per capita amount of purchased antibiotics prescribed by the GP, in the previous 12 months
	participation rate of mammography in the previous 24 months among 45- to 65-year-old women
	participation rate of cervical cytology in the previous 36 months among 25- to 65-year-old women
all-cause premature mortality	mortality rate for adults 18–64 years old who had not changed GMPs in the five years prior to the investigated year

**Table 2:** Service delivery rates as pathway indicators for adults living in segregated Roma settlements (SRSs) and living in the same municipalities’ complementary areas (non-SRSs) with the age- and sex-standardized relative service delivery rates (RRs) for inhabitants of SRSs.

Indicators	Crude Rates in the Sample, N (%)	SRS		Non-SRS		RR [95% CI]
		N	Standardized Rates [95% CI]	N	Standardized Rates [95% CI]	
number of GP appointments (appointments per capita per year)	454,257 (8.31)	22,322	1.144 [1.129–1.159]	431,935	0.994 [0.991–0.996]	1.152 [1.136–1.167]
number of subjects receiving outpatient care at least once a year (%)	40,255 (73.62)	2059	0.97 [0.929–1.012]	38,196	1.002 [0.992–1.012]	0.968 [0.926–1.012]
number of interventions in outpatient care a year (interventions per capita)	1,834,015 (33.54)	76,715	0.897 [0.891–0.904]	1,757,300	1.005 [1.004–1.007]	0.893 [0.886–0.899]
reimbursement in outpatient care a year (EURO per capita)	2,011,662 (36.79)	87,311	0.924 [0.918–0.930]	1,924,351	1.004 [1.002–1.005]	0.920 [0.914–0.927]
number of subjects having imaging examination at least once a year (%)	28,629 (52.36)	1556	1.061 [1.009–1.115]	27,073	0.997 [0.985–1.009]	1.064 [1.011–1.120]
number of imaging examinations a year (examinations per capita)	1,076,117 (19.68)	44,413	0.899 [0.891–0.908]	1,031,704	1.005 [1.003–1.007]	0.895 [0.887–0.904]
reimbursement for imaging examination a year (EURO per capita)	703,974 (12.87)	34,139	1.034 [1.023–1.046]	669,835	0.998 [0.996–1.001]	1.036 [1.025–1.047]
number of subjects being hospitalized at least once a year (%)	9007 (16.47)	522	1.269 [1.165–1.383]	8,485	0.987 [0.966–1.008]	1.286 [1.177–1.405]
duration of hospitalization a year (days per capita)	142,475 (2.61)	58,265	1.162 [1.132–1.192]	136,649	0.994 [0.989–0.999]	1.168 [1.138–1.200]
reimbursement in inpatient care a year (EURO per capita)	7,554,882 (138.16)	299,580	1.058 [1.054–1.061]	7,255,301	0.9977 [0.9970–0.9984]	1.060 [1.057–1.064]

**Table 3:** Service delivery rates as performance indicators for adults living in segregated Roma settlements (SRSs) and living in the same municipalities’ complementary areas (non-SRSs) with the age- and sex-standardized relative service delivery rates (RRs) for inhabitants of SRSs.

Indicators	Crude Rates in the Sample, N (%)	SRS		Non-SRS		RR [95% CI]
		N	Standardized Rates [95% CI]	N	Standardized Rates [95% CI]	
influenza vaccination, above 65 years of age	2523 (23.22)	29	0.679 [0.472–0.977]	2494	1.006 [0.967–1.046]	0.675 [0.468–0.973]
prevalence of hypertension, 40–54 years	3461 (25.57)	200	0.999 [0.870–1.148]	3261	1.000 [0.966–1.035]	0.999 [0.866–1.153]
prevalence of hypertension, 55–69 years	6938 (55.99)	200	0.916 [0.798–1.053]	6738	1.003 [0.979–1.027]	0.914 [0.794–1.052]
Serum-creatinine assessment, hypertension	10447 (60.99)	309	0.964 [0.863–1.078]	10,138	1.001 [0.982–1.021]	0.963 [0.860–1.079]
lipid status check-up, hypertension and/or diabetes	9666 (54.73)	324	1.067 [0.957–1.189]	9342	0.998 [0.978–1.018]	1.069 [0.957–1.194]
beta–blocker, ischemic heart diseases	523 (51.73)	23	1.049 [0.697–1.579]	500	0.998 [0.914–1.089]	1.052 [0.692–1.597]
HbA1c check-up, diabetes	2674 (73.64)	82	0.817 [0.658–1.015]	2592	1.007 [0.969–1.047]	0.811 [0.651–1.011]
ophthalmologic check-up, diabetes	1313 (36.16)	34	0.701 [0.501–0.982]	1279	1.011 [0.958–1.068]	0.693 [0.493–0.975]
prevalence of diabetes, 40–54 years	618 (4.57)	40	1.123 [0.824–1.531]	578	0.992 [0.915–1.077]	1.132 [0.821–1.559]
prevalence of diabetes, 55–69 years	1241 (10.01)	36	0.929 [0.670–1.287]	1205	1.002 [0.947–1.061]	0.927 [0.665–1.291]
treatment with antibiotics	13371 (24.49)	570	0.791 [0.728–0.858]	12,801	1.012 [0.995–1.030]	0.781 [0.718–0.850]
mammography, 45–64 years	4451 (47.96)	76	0.392 [0.313–0.490]	4375	1.028 [0.998–1.059]	0.381 [0.304–0.478]
cervix cytology, 25–64 years	7365 (40.22)	428	0.959 [0.873–1.055]	6937	1.003 [0.979–1.027]	0.957 [0.868–1.055]
